# Incidence and risk factors for postoperative sore throat after general anesthesia with endotracheal intubation: prospective cohort study

**DOI:** 10.1097/MS9.0000000000000786

**Published:** 2023-05-17

**Authors:** Zenebe Bekele, Zewde Melese

**Affiliations:** Department of Anesthesia, Ambo University, Ambo, Ethiopia

**Keywords:** endotracheal tube, sore throat following surgery

## Abstract

Postoperative sore throat (POST) is a common, unpleasant, and upsetting complication of tracheal intubation that increases postoperative morbidity and causes patient dissatisfaction after general anaesthesia.

**Objective:**

To determine the prevalence and risk factors of POST following endotracheal intubation under general anaesthesia.

**Materials and methods:**

From 20 April to 20 May 2021, a multicenter prospective cohort study design and systematic random sampling were used to select study participants from patients over the age of 18 who underwent anaesthesia-assisted surgery with endotracheal intubation. A structured questionnaire was developed after reading some of the relevant literature. SPSS version 20 was used to enter and analyze the data, and binary logistic regression was used to select a variable for multivariable logistic regression. To identify statistically significant factors, a *P* value of 0.05 for the association between the independent and dependent variables was used in multivariable analysis.

**Results:**

Sore throat occurred in 94 (61.8%) of the 152 patients who had undergone surgery under general anaesthesia with endotracheal intubation. The size of the endotracheal intubation was found to have a *p* value of 0.04, (adjusted odds ratio 0.04, 95% CI 0.002–0.79), and the duration of the anaesthesia was found to have a *p* value of 0.003, (adjusted odds ratio 4.5, 95% CI 1.66–12.18). The POST was associated with a large endotracheal tube, patient age, and an extended period of anaesthesia in this study, with a prevalence of 94 (61.8%) cases.

**Conclusion and recommendation:**

In this study, the incidence of POST was high 94 (61.8%), large size endotracheal tube, patient age, and duration of anaesthesia were associated factors for POST. Awareness creation through training based on research findings should be made about the problem for all health professionals who were involved in postoperative patient care.

## Introduction

Highlights

- The incidence of postoperative sore throat (POST) in this study was 61.8%.
- Size of endotracheal tube, duration of anaesthesia and age were risk factor for POST.
- POST is listed from the top as the patients’ most undesirable outcome

Endotracheal intubation frequently results in sore throats, which can be highly upsetting for the patient and cause sleep disruptions and unpleasant recollections^1^. The tracheal mucosa has been observed to release inflammatory mediators after intubation, suggesting that the aetiology of postoperative sore throat (POST) is likely an inflammatory process, uncertainty exists regarding the precise anatomical location of neck pain in patients^2,3-5^.

Most research studies report varying rates of POST, but some claim the rate is higher after general anaesthesia, this high variability is caused by a number of factors, including the type of airway device used, the technique of insertion, use or type of lubricant, cuff pressure, length of procedure, and anaesthesia, agent used, evaluation methods, and a variety of patient characteristics^6–9^.

POST complaints can range in severity from slight throat irritation to incapacitating discomfort that prevents swallowing, and transitory voice alterations are frequently noticed during the postoperative visit, while rarely delaying release, these concerns nonetheless have an impact on patient satisfaction and may limit their ability to engage in certain activities after leaving the hospital, the recovery from a pharyngolaryngeal damage can often take months, and in certain situations, it may even be permanent, in many circumstances, the interference can result in trauma, foreign body contamination, mucosal dryness, and airway irritation, all of which might present in different ways in the postoperative period^10–13^.

Many different methods, both non-pharmacological and pharmacological, have been explored to lessen POST, with varying degrees of success. Non-pharmacological techniques include using a smaller endotracheal tube (ETT), applying water-soluble gel to the ETT, being careful during airway instrumentation, waiting until complete relaxation before intubation, performing gentle oropharyngeal suction, reducing intra-cuff pressure, minimizing cuff inflation, and deflating the tracheal tube completely. These methods have been found to reduce the likelihood of POST. Pharmacological measures, such as using lignocaine, steroids, non-steroidal anti-inflammatory drugs, and gargling with azulene sulfonic acid, have also been shown to decrease the incidence of POST^5–12^.

Several studies have been conducted globally on the prevalence of POST and its risk factors for POST and risk factors are numerous^6–9^. Much research was done worldwide regarding the incidence of POST and its associated risk factors are/is overwhelming. For a long time, few studies have been performed to determine the incidence of POST and to find measures for its prevention, most of these Studies have been done in developed and limited studies have been done on predictors of POST in developing countries where resources and qualified man powers are limited^5,14^. Therefore, this research aimed to assess the magnitude and associated factors of POST, and this research will help to minimize combinations of risk factors reduce the incidence, severity of POST, raise awareness of the variables associated with an increased incidence of POST and increase postoperative patient satisfaction.

## Method and material

### Study area

This study was conducted at Ambo University Referral Hospital and Ambo General Hospital, which is located in the West Shoa Zone, Oromia region of Ethiopia, about 114 Km from Addis Ababa. The hospital is serving more than 2 million people in the catchment area. Specialty surgical units, internal medicine, paediatrics, gynaecology and obstetrics, ear nose throat, and other minor departments are among the many healthcare services offered by the hospital. The hospital was supposed to serve an estimated 3.5 million people, and perform an average of 1800 surgeries each year. Orthopaedic, maxillofacial, dental, ophthalmology, general surgery, gynaecologic, and obstetric specialty units performed the majority of surgeries. Registered in the Research Registry and UIN= 8169 and the methodology in this study has been reported according to STROSS guidelines 2021^3,4^.

Study period: The study was conducted from 20 April to 20 May 2021.

Study design: Prospective cohort

## Source population

All patients who underwent surgery under general anaesthesia with endotracheal intubation

## Study population

Patients who underwent surgery under general anaesthesia with endotracheal intubation who met inclusion criteria during the study period

## Eligibility criteria

### Inclusion criteria

American Society of Anesthesiology (ASA) classification I, II, and ages 18 and above performed under endotracheal intubation were included in the study.

### Exclusion criteria

Patients whose surgery is performed in the neck, mouth, or throat area, recent or ongoing upper respiratory infection, double lumen tubes, and existing preoperative sore throat cases were excluded from the study.

## Study variables

### Dependent

POST (yes or no)

#### Independent

Age, sex, body mass index, the urgency of the case, ETT size, use of airways, ASA classification, duration of anaesthesia, number of attempts during laryngoscopy and intubation, diagnosis of the patient, and mallampathy class of pt.

## Sample size and sampling technique

### Sample size

The sample size was determined by taking the following assumptions: the incidence of POST was found 46% in Black lion specialized hospital, Addis Ababa, Ethiopia. [16], a confidence interval of 95% and a margin of error of 0.05. The sample size was determined using the following single population proportion formula.

The used Formula was:

*n*=*Z* ^2^ (*P*) (1−*p*)/*d* ^2^= = 382

Whereas;

*n*= sample size required

*Z*=the standard normal variable with 95% CI (1.96)

*P* = Prevalence (46%)

*d* = margin of sampling error to be tolerated = 5% (0.05)

To get the sample size with confidence interval of 95% and margin of error 5%,

However; the total population who was admitted within the study period was less than 10 000 so adjustment formula was used by applying a finite population correction formula, the sample size was calculated as;

*nf*=*n*/ (1+*n*/*N*)

Were *n*= the minimum sample size =382

*N*=Total number of patients underwent surgery =215

*nf*=the final required sample size (adjusted sample size).

Therefore, *nf*= 382/ (1+382/215) =138.

By adding non respondents rate=10%, 138×10/100=14+138 =152 was calculated sample size

Three months’ patient report who underwent surgery were 645 in AURH and 390 in AGH from report.so the size of one month was 215 and 130, respectively, and was 95 participants from AURH and 57 from GH that became final sample size of 152 from both hospitals.

To get the study participants of two hospitals (AURH and AGH), it is calculated as: *ni*=(*n*/*N*) ×*Ni*

Where *ni* is the required number of sample size of each hospital, *n* is the total calculated sample size, *N* is the total surgery of both hospitals.

*Ni* is the total surgery size of each hospital.

For;-

xx: *ni*=(*n*/*N*) ×*Ni*; *ni*=152 ×130/345 = 57
xxx: *ni*=(*n/N*) ×*Ni* ; *ni*=152×215/345 = 95

### Sampling technique

Systematic random sampling technique was used to select study participants. The calculated sample size was 152. So, sampling interval (*k*) was calculated as follows.

*K*=*N*/*n* where, *n* = total sample size

345/152= 2 for both AURH and AGH *k*=sampling interval

*N* = Total study population

Therefore, 2 was the sampling interval and the first study participant was selected from the first two cases of the list by lottery method.

The first study participant was the second case from the list of surgery and the consequent cases would be 4, 6, and 8, up to 345. for example.

**Table:** 

1	2	3	4	5	6	7	8	9	---345

### Data collection technique and instrument

Data were collected using questionnaires prepared in English and then translated into Afan Oromo. Data were collected by two trained anaesthetist and follow-up was made for every patient postoperatively for 24 h. Number of attempts during laryngoscopy to intubate the trachea, size of ETT employed, duration of anaesthesia, demographic variables, and overall medical condition of the patients were recorded.

### Data quality control

Pretests were made in one of West Shoa hospitals on 5% of the participants, orientation and training about the objective and process of data collection were given to two Anaesthetists before data collection. Cross-checking was made during data collection by the supervisor.

## Data analysis and interpretation

The data were coded and entered into the SPSS version 20 statistical package. All independent variables were analyzed by binary logistic regression with the dependent variable POST and those with a *p* value less than or equal to 0.2 from the bivariate analysis were fitted to multivariate logistic regression to verify their association with the outcome variable POST. The odds ratio, 95% CI, and *p* value were calculated to identify associated factors and to determine the strength of the association. A *p* value of 0.05 was considered statistically significant. The Hosmer–Lemeshow test of goodness of fit was performed to check the suitability of the model for the analysis.

## Operational definition

POST: Within 24 h. of the surgery, patients felt of either pain, discomfort, or both during swallowing.

## Ethical consideration

Ethical clearance was obtained from the Ethical review committee of the college of health science, at Ambo Univeristy Referral Hospital and the letter was given to xxx and xxx for support and permission. Permission was obtained from the Anaesthesia department after a clearance letter was submitted to conduct the research. Informed verbal consent was obtained from respondents after giving them information about the study. In addition, all the responses were kept confidential and anonymous.

## Result

### Sociodemographic characteristics of study participants

The study was conducted on total of 152 patients who underwent surgery by general anaesthesia with endotracheal intubation. In this study 150 (98.7%) of the respondents age were between 18–65 years, 107 (70.4%) were female (Table 1).

**Table 1:** Socio-demographic characteristics of study+ participants (*N*=152)

Variables		Frequency	Percent (%)
Age	18–65 years	150	98.7
	>65 years	2	1.3
Sex	Male	45	29.6
	Female	107	70.4
BMI	<18.5	42	27.6
	18.5–24.9	88	57.9
	25–29.5	22	14.5

### Anaesthesia and surgery related characteristics of study participant

The majority of patients 118 (95.9%) were underwent elective surgery. The majority of the study participants, 112 (73.7%) were underwent general surgery procedures. Among the participants 59 (38.8%) were intubated with a 6.5 mm tube, 23 (15.1%) were intubated with 6.0 ETT size, and 130 (85.5%) were intubated by laryngoscopy size number three. The duration of anaesthesia and surgery in the study was 1.57 min mean duration. In the study period, the minimum duration that the patients stayed under anaesthesia was 1 h, and the maximum was 3 h. This study also showed that *N*= 22(14%) of the patients stayed more than 2 h *N*=42(28%) of the patients stayed 1–2 h and *N*=88(58%) of the patients stayed less than 1 h under anaesthesia (Table 2).

**Table 2:** Anesthetic and surgery related characteristics of study participants (*N* = 123)

Variables		Frequency	Percent (%)
Diagnosis	Surgery case	62	40.8
	Gynaecologic case	13	8.6
	Obstetric case	72	47.4
	orthopaedics case	5	3.3
Urgency of cases	Elective	112	73.7
	Emergency	40	26.3
Mallampati classification	Class 1	54	35.5
	Class 2	95	62.5
	Class 3	3	2
ETT size	5.5 ID	4	2.6
	6.0 ID	23	15.1
	6.5 ID	59	38.8
	7.0 ID	10	6.6
	7.5 ID	56	36.8
Size of laryngoscope blade	Number 3	130	85.5
	Number 4	22	14.5
Size of NGT	16F	1	3.9
	18F	4	2.6
Duration of Anaesthesia	<1 h	88	57.9
	1–2 h	42	27.6
	>2 h	22	14.5
ASA classification	ASA I	100	65.8
	ASA II	52	34.2

ASA, American society of Anesthesiology; ETT, endotracheal tube; NGT, naso gastric tube.

### Incidence of POST of the study participant

**Figure 1:**
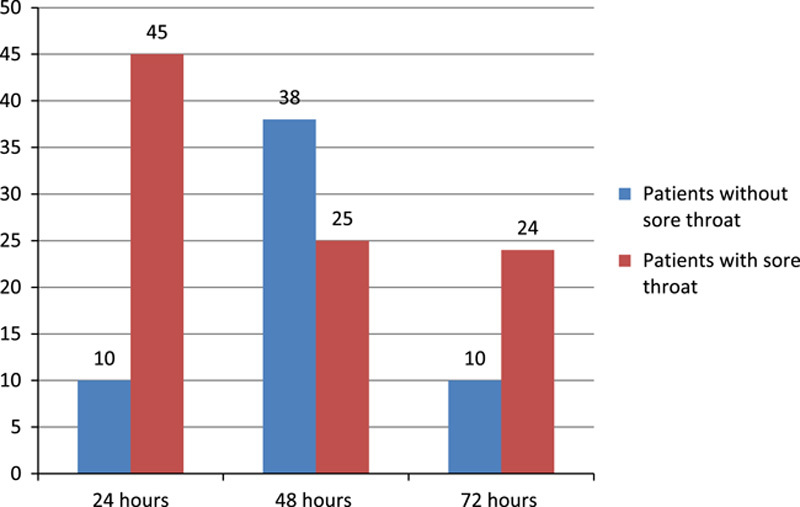
Proportional allocation of study participants for each hospital.

The incidence of POST in this study was 94 (61.8%)The highest occurrence of POST was within 6 h which were 45% followed by 25% within the next 12 h and 24% within 24 h (Fig. 1).

### Factors associated with POST in bivariate analysis

On bivariate analysis age, sex, size of endotracheal tube, duration of anaesthesia, ASA classification, and urgency of surgery were associated factors for POST.

### Multivariate analysis result of factors associated with POST

Duration of anaesthesia *p* value of 0.003, adjusted odds ratio= 4.5, 95% CI: (1.66–12.18). age older than 65 with *p* value less than 0.001, (adjusted odds ratio= 3.5,95% CI; (1.03–1.1) were associated factors for POST and this study also revealed that larger size of ETT was associated with the occurrence of the POST with *p* values of 0.03, 0.029, and 0.001for ETT sizes of 6.5, 7.0, and 7.5, respectively (Table 3).

**Table 3:** Factors associated with postoperative sore throat of patients who underwent general anaesthesia with endotracheal intubation (*N*=152)

		POST				
Variables		Yes (*n*)	No (*n*)	COR (95% CI)	AOR (95% CI)	*P*
Age	18–65	43	106	1	1	
	>65	2	1	4.9 (1.03–1.1)	3.5 (1.03–1.1)	0.04
Sex	M	17	28	1	1	
	F	29	78	0.61 (0.29–1.28)	1.63 (0.78–3.42)	0.19
Size of ETT	6.0	14	29	1	1	
	6.5	24	21	3.2 (1.21, 8.232)	3.8 (1.4, 10.7)	0.03
	7.0	20	15	4.2 (1.46, 11.91)	3.6 (1.149, 11.2)	0.029
	7.5	16	11	4.6 (1.42, 15.08)	4.1 (1.140, 14.29)	0.001
Duration of anaesthesia	≤2 h	37	93	1	1	
	>2 h	11	11	2.33 (0.45–3.76)	4.5 (1.66–12.18)	0.003
ASA classification	ASA I	46	54	1	1	
	ASA II	31	21	0.54 (0.37–0.78)	3.43 (0.3–39.64)	0.32
Urgency of case	Elective	15	25	1	1	
	Emergency	31	81	1.57 (0.73–3.36)	0.64 (0.3–1.37	0.25

Where: 1=reference group.

AOR, adjusted odd ratio; ASA, American society of Anesthesiology; COR, crude odd ratio; ETT, endotracheal tube; n, number; POST, Postoperative sore throat.

## Discussion

POST is a common, uncomfortable, and distressing consequence of tracheal intubation, contributing to postoperative morbidity and patient dissatisfaction following general anaesthesia^15^.

In our study, the incidence of POST was 61.8%, comparable to the cross-sectional study by Melkamu and colleagues in Gondar with 240 participants^16^. But our results were better than the study conducted at Toronto Western Hospital. 12.1% said they had a sore throat^13^. The former is performed in a country with limited resources, where there is an imbalance between demand and supply that makes it impossible for an anaesthetist to select different ETT sizes for different age categories; Therefore, using an improper size contributes to damage to the larynx mucosa, which causes POST, again, soaking the ETT tubing in the detergents for recycling purposes can also contribute to POSTs due to the lack of ETTs^3–6^.

This study also showed that there was a significant association between being over 65 and the occurrence of POST, similar to a cross-sectional study of 97 participants by Jansson and colleagues^17^. This can be age related and can cause the oral mucosa to become thin, smooth and dry with a loss of elasticity and stippling. These changes are likely the result of changes in the epithelium and dermis, reduced proliferative activity of fibroblasts, proteoglycan synthesis and protein and collagen synthesis^18^. Age-related changes in the oral mucosa can lead to POST^7,19–23^. In contrast to this study, however, research conducted at Black Lion Hospital^21^ and a cross-sectional study by Fenta *et al*.^8^ found in 123 patients that age was not a significant factor for POST. This difference may be due to the existence of a protocol for selecting an appropriately sized ETT and the accessibility of using the calculated ETT size for all age categories at their study site.

This study also showed a significant association between the duration of anaesthesia longer than 2 h, 11 (7.2%) of the patients staying longer than 2 h. Develop POST. Compared with a study conducted at Watford General Hospital, this result is low at 63.9% for anaesthesia lasting more than 90 min^14^. In another study conducted at Black Lion Hospital, 52 (45.6%) of the study participants complained of various forms of POST, showing that 84.2% of the patients remained under anaesthesia for more than 90 minutes., in our study, these lower scores in our study may be due to only performing surgeries that do not take long, while major surgeries that take longer, such as neurosurgery, were included in the Black Lion Hospital study.

In this study, ETT size was an associated factor for POST, comparable to studies by Efrem Fenta in 123 participants and Maria Jansson in 97 participants^24,25^, tension fold in the tracheal mucosa causing direct trauma to the tracheal mucosa and potentially leading to POST.

### Limitation of the study

In this study, the presence of a blood-stained tracheal tube on extubation, tracheal intubation without neuromuscular blockade, high tracheal tube cuff pressures, the duration of extubation, coughing during extubation, and suctioning before extubation, awake or deep extubation, timing of suctioning, suction pressure, and need for airway manoeuvres immediately following extubation and emergency surgery might bias the result hence it was not addressed in study.

## Conclusion

In this study the incidence of POST was high. Geriatric age group, ETT size, and procedures lasting greater than or equal to 2 h were the independent risk factors for POST.

### Recommendation

Awareness creation through training based on research finding should be made about the problem for all health professionals who will be involved in patient management after the operation. Hospital should have protocol on the way of ETT tube usage, and hospitals should provide adequate amount and different size of ETT.

We also recommend further research in this area undressing, the presence of a blood-stained tracheal tube on extubation, tracheal intubation without neuromuscular blockade, high tracheal tube cuff pressures, the duration of extubation, coughing during extubation, and suctioning before extubation, awake or deep extubation, timing of suctioning, suction pressure, and need for airway manoeuvres immediately following extubation and emergency surgery.

## Ethical approval

Ethical approval was secured from Ambo University institutional review board.

## Consent

No individual and sensitive data and data are available on request.

## Source of funding

None.

## Author contribution

Z.M. and Z.B. have made substantial contributions to conception, writing—review and also contributed in editing of the manuscript drafts for scientific merit and depth.

## Conflicts of interest disclosure

None.

## Provenance and peer review

Not commissioned, externally peer-reviewed.

## Acknowledgements

The authors acknowledge Jemal Suleiman, Kasahun Girma, Megersa Benti and Yeshitila Sintayehu for their participations.
